# Vertical transmission of *Anaplasma platys* and *Leishmania infantum* in dogs during the first half of gestation

**DOI:** 10.1186/s13071-016-1545-y

**Published:** 2016-05-10

**Authors:** Maria Stefania Latrofa, Filipe Dantas-Torres, Donato de Caprariis, Cinzia Cantacessi, Gioia Capelli, Riccardo Paolo Lia, Edward B. Breitschwerdt, Domenico Otranto

**Affiliations:** Dipartimento di Medicina Veterinaria, Università degli Studi di Bari, Valenzano, Italy; Aggeu Magalhães Research Centre, Oswaldo Cruz Foundation, Recife, Pernambuco Brazil; Department of Veterinary Medicine, University of Cambridge, Cambridge, UK; Istituto Zooprofilattico Sperimentale delle Venezie, Legnaro, Padova Italy; Intracellular Pathogens Research Laboratory, Center for Comparative Medicine, College of Veterinary Medicine, North Carolina State University, Raleigh, NC USA

**Keywords:** Vertical transmission, *Leishmania infantum*, *Anaplasma platys*, Dog, Real-time PCR

## Abstract

**Background:**

*Leishmania infantum* is a canine zoonotic vector-borne protozoan pathogen transmitted by phlebotomine sand flies, whereas *Anaplasma platys* is a bacterium most likely transmitted by ticks. While vertical transmission of *L. infantum* from pregnant bitches to their offspring has been documented, thus far no studies have explored the possibility of vertical transmission of *A. platys* in dogs. This study investigated the occurrence of vertical transmission of *L. infantum* and *A. platys* in sheltered dogs during the first half of gestation, in an area of southern Italy characterised by a high incidence of infection by both pathogens.

**Methods:**

The study population included 20 bitches (*n* = 10 pregnant, at 25–35 days of pregnancy; *n* = 10 non-pregnant), all subjected to ovariohysterectomy, which were examined for the presence of *L. infantum* and *A. platys* via cytological screening of bone marrow and whole blood samples*.* Infection by *L. infantum* and *A. platys* was also tested by immunofluorescence antibody test (IFAT) and quantitative real-time PCR (qPCR) targeting both pathogens. Selected tissue samples (*n* = 210) collected during surgical procedures from bitches and foetuses (*n* = 20) were assessed for the presence of *L. infantum* and *A. platys* by qPCR targeting a fragment of the kinetoplast minicircle DNA (kDNA) and the 16S rRNA gene, respectively.

**Results:**

*Leishmania infantum* DNA was not amplified from either uteri or ovaries from pregnant bitches or foetal tissue samples, whereas a subset of ovarian (*n* = 2) and uterine (*n* = 4) tissue samples from non-pregnant bitches were infected, with parasite loads of up to 3.09 × 10 and 7.51 parasite/PCR reaction, respectively. Conversely, uterine (*n* = 10) and ovarian (*n* = 8) tissues from both pregnant and non-pregnant bitches, together with a subset (*n* = 5) of foetal tissue samples were qPCR positive for *A. platys. Leishmania infantum* and *A. platys* nucleic acids were amplified from two uteri from non-pregnant bitches, with parasite loads of up to 2.32 × 10^-3^ and 2.05 parasite/per PCR reaction, respectively.

**Conclusions:**

Results from this study suggest that, in contrast to *L. infantum*, *A. platys* can be transmitted from pregnant dogs to their offspring during the first half of gestation. This hypothesis remains to be verified, for instance via direct observation of parasites in postpartum foetal tissues.

## Background

*Leishmania infantum* (Kinetoplastida, Trypanosomatidae) and *Anaplasma platys* (Rickettsiales, Anaplasmataceae) are the causative agents of canine leishmaniosis (CanL) and canine infectious cyclic thrombocytopenia (CICT), respectively [1]. While *L. infantum* is primarily transmitted by phlebotomine sand flies of the genera *Phlebotomus* and *Lutzomyia* (Diptera, Psychodidae) [2], alternative modes of transmission have been investigated. These include blood transfusions [3, 4], as well as sexual [5, 6] and vertical transmission [7–12]. In particular, the latter has been hypothesized to occur in naturally infected dogs, resulting in both stillborn and live puppies [7–10]. Vertical transmission of *L. infantum* has been suggested to represent an important mechanism for the dissemination and maintenance of this pathogen, especially in the (apparent) absence of known competent vectors in non-endemic areas [10, 12–14]. For instance, a high prevalence of infection (i.e. 20 %) has been recorded in dogs from an area of the United States where the presence of phlebotomine sand fly vectors is yet to be confirmed [9, 10]. *Rhipicephalus sanguineus* (*sensu lato*) (Acari, Ixodidae), also referred to as the ‘brown dog tick’ or ‘kennel tick’, has been indicated as a potential vector of *A. platys,* mainly as a consequence of the detection of bacterial DNA in this arthropod species [15–18]. However, the role of ticks in the transmission of this bacterium has yet to be fully proven. Similarly, and to the best of our knowledge, thus far*,* no studies have assessed the potential occurrence of vertical transmission of *A. platys* in dogs, or of vertical transmission of *L. infantum* during the first half of gestation. Here, we explored the potential occurrence of vertical transmission of *L. infantum* and *A. platys* in naturally infected dogs, during early gestation, from a hyperendemic area for both pathogens.

## Methods

### Ethics statement

Animals involved in this study were enrolled in a sterilization program in 2008 and 2011 and were part of a previous study for the prevention and control of canine vector borne diseases [19]. The study above was carried out according to the guidelines for Good Clinical Practice (GCP GL9 VICH, 2000) and authorized by the Italian Ministry of Health (authorization number 72/2009C, n°. 69062; 11/28/2008).

### Animals

All animals enrolled in the present study were housed in a private dog shelter in Putignano, province of Bari, Apulia region, Italy, where the incidence of *L. infantum* (i.e. 47.6 %) and *A. platys* (i.e. 17.2 %) infections have previously been determined [19–21]. Twenty asymptomatic mixed-breed bitches were screened for infection by *L. infantum* and *A. platys* by whole blood and bone marrow cytological examination. In order to confirm the infection status, dogs were also tested by indirect immunofluorescent antibody test (IFAT) on whole blood (*L. infantum*) and pathogen DNA amplification from whole blood, bone marrow and skin samples (both *L. infantum* and *A. platys*) (Table 1). All bitches were subjected to clinical and abdominal ultrasound examinations for verification of the pregnancy status, followed by an ovariohysterectomy. Ten dogs (i.e. nine positive and one negative control) (aged from 7 months to 1 year old) were from 25 to 35 days pregnant (Table 1). Following surgery, two foetuses were randomly collected from the uteri of each pregnant bitch (Table 2) and foetal crown-rump length was measured to confirm their gestational age [22]. The remaining (age-matched) 10 dogs (i.e. nine positive and one negative control) enrolled in the study were non-pregnant bitches (Table 1).

**Table 1:** Distribution of *Leishmania infantum* and *Anaplasma platys* infections in different tissue samples from pregnant and non-pregnant bitches (Pos/Tot, %)

Pathogen	Dogs		Blood	Bone marrow	Skin	Uterus	Ovary	Placentae	Total
			Pos/Tot	Pos/Tot	Pos/Tot	Pos/Tot	Pos/Tot	Pos/Tot	Pos/Tot (%)
*L. infantum*									
	Pregnant bitches	(*n* = 9)	1/9	7/9	9/9	0/9	0/9	1/9	18/54 (33.3)
	Non-pregnant bitches	(*n* = 9)	2/9	5/9	4/9	4/9	2/9	–	17/45 (37.8)
	Total Pos/Tot (%)		3/18 (16.7)	12/18 (66.7)	13/18 (72.2)	4/18 (22.2)	2/18 (11.1)	1/9 (11.1)	35/99 (35.3)
*A. platys*									
	Pregnant bitches	(*n* = 9)	3/9	4/9	3/9	3/9	2/9	1/9	16/54 (29.6)
	Non-pregnant bitches	(*n* = 9)	0/9	4/9	1/9	7/9	6/9	–	18/45 (40)
	Total Pos/Tot (%)		3/18 (16.6)	8/18 (44.4)	4/18 (22.2)	10/18 (55.5)	8/18 (44.4)	1/9 (11.1)	34/99 (34.3)

**Table 2:** Distribution of *Leishmania infantum* and *Anaplasma platys* infection in foetal tissue from naturally infected pregnant bitches. Starting Quantity (SQ) mean value of parasite load per PCR reaction

Foetuses	Bone marrow		Liver		Spleen		Amniotic fluid		Umbilical cord	
	*L. infantum* (SQ mean)	*A. platys* (SQ mean)	*L. infantum* (SQ mean)	*A. platys* (SQ mean)	*L. infantum* (SQ mean)	*A. platys* (SQ mean)	*L. infantum* (SQ mean)	*A. platys* (SQ mean)	*L. infantum* (SQ mean)	*A. platys* (SQ mean)
820634 A	–^b^	–	–	2.83 × 10^-2^	–	–	–	–	–	–
B				3.5 × 10^-2^						
821717 A	–	–	–	–	–	–	–	–	–	–
B										
828965 A	–	–	–	–	–	1.65 × 10^-1^	–	–	–	–
B						5.25 × 10^-2^				
821240 A	–	–	–	–	–	6.52 × 10^-2^	–	–	–	–
B										
823993 A	–	–	–	–	–	–	–	–	–	–
B										
42686 A	–	–	–	–	–	–	–	–	–	–
B										
43077 A	–	–	–	–	–	–	–	–	8.14 × 10^-4^	–
B										
42783A	–	–	–	–	–	–	–	–	–	–
B										
43055 A	–	–	–	–	–	–	–	–	–	–
B										
42825 A^a^	–	–	–	–	–	–	–	–	–	–
B										

^a^Dog used as negative control; ^b^tissue qPCR negative for pathogen DNA

### Sample collection and diagnostic procedures

At the time of ovariohysterectomy, amniotic fluid and tissues samples (*n* = 210) including whole blood, skin, uterus, ovaries, placenta, liver, spleen, umbilical cord and bone marrow were collected from individual bitches and foetuses, in sterile conditions, and stored at -20 °C until extraction of genomic DNA (Tables 1 and 2). Whole blood samples (4 ml) were collected from the brachial or jugular veins of bitches and aliquots (2 ml) were centrifuged at 1678 *g* for 10 min, the sera were separated and stored in individually labelled Eppendorf tubes at -20 °C until tested. An IFAT was performed using promastigotes of *L. infantum* zymodeme MON1 as antigen [19] to investigate previous exposure to *Leishmania* parasites. While under general anaesthesia, bone marrow samples were obtained from bitches by aspiration from the iliac crest using Rosenthal needles (16 or 18 Gauge) and stored at -20 °C in individual tubes with 1 ml of phosphate buffered saline (PBS) until subsequent testing. Aliquots of whole blood and bone marrow samples were smeared on slides and microscopically examined by staining with May-Grünwald-Giemsa Quick Stain (Bio Optica Spa, Italy). Skin tissue samples of approximately 0.5 cm^2^ were collected from the interscapular region of bitches and processed as described previously [19]. Uterus, ovaries and placenta biopsy samples were collected and stored in individual sterile tubes (Table 1).

Amniotic fluid (1 ml) was collected for each foetus from the intact uterus using a sterile needle, and stored in individual tubes. Umbilical cord, spleen and liver samples (1 cm^2^) were collected by a sterile scalpel and stored in tubes containing PBS. The bone marrow samples were collected by cutting the tip of the sternum and stored in tubes with 1 ml of PBS (Table 2).

### DNA extraction and parasite load determination

Total DNA was extracted from whole blood, skin, uterus, ovaries, placenta, liver, spleen and umbilical cord samples using the Genomic DNA Purification Kit (Gentra Systems, Minnesota, USA), while genomic DNA from amniotic fluid and bone marrow samples was extracted using QIAampDNA Micro Kit (Qiagen, GmbH, Hilden, Germany). For each sample, two qPCR reactions were individually performed for the detection and quantification of *L. infantum* and *A. platys* nucleic acids, using primers and probes targeting, respectively, the kinetoplast minicircle DNA (kDNA) and 16S rRNA gene, as described previously [18, 23]. DNA extracted from lymph nodes and whole blood from *L. infantum* and *A. platys*-infected dogs were included as positive controls. Quantification of DNA of *L. infantum* and *A. platys* was performed using a 10-fold dilution series of standard DNA from promastigotes (log phase concentration, 1.7 × 10^6^ parasites/ml) of *L. infantum* (zymodeme MON-1) and from *A. platys*-infected blood with a concentration of 5.6 × 10^5^ infected platelets/100 μl. The detection limits of the qPCRs were assessed using serial dilutions from 1.7 × 10^-2^ to 1.7 × 10^-7^ parasites (*L. infantum*) and from 2.24 × 10^2^ to 2.24 × 10^-6^ infected platelets (*A. platys*) per reaction (2 μl of DNA template), respectively.

## Results

Dogs included in the study, with the exception of negative controls, were serologically positive for *L. infantum* (IFAT titre up to 1:160) and for *A. platys* by pathogen DNA amplification from whole blood, bone marrow and skin samples (both *L. infantum* and *A. platys*) (Tables 1, 3 and 4). Eighteen out of 54 (33.3 %) tissue samples collected from pregnant bitches were positive to *L. infantum* by qPCR; of these, only one placental tissue sample was positive for this parasite, at a detected load of 1.2 × 10^-3^/PCR reaction (Tables 1 and 3). No uterine and ovarian samples were positive for *L. infantum* (Tables 1 and 3). Of the 100 foetal samples collected, only one umbilical cord was *L. infantum*-positive, albeit with a low parasite load of 8.14 × 10^-4^/PCR reaction (Table 2). Conversely, 17/45 (37.8 %) tissue samples collected from non-pregnant bitches were qPCR positive for *L. infantum* (Tables 1  and 4), with the highest parasite load detected in ovarian and uterine samples (i.e. up to 3.09 × 10 and 7.51/PCR reaction, respectively) (Table 4).

**Table 3:** Real-time PCR detection and quantification of *Leishmania infantum* and *Anaplasma platys* DNA. in tissue samples from pregnant bitches. Starting Quantity (SQ) mean value of parasite load per PCR reaction

Dogs	Blood		Bone marrow		Skin		Uterus		Ovary		Placentae	
	*L. infantum* (SQ mean)	*A. platys* (SQ mean)	*L. infantum* (SQ mean)	*A. platys* (SQ mean)	*L. infantum* (SQ mean)	*A. platys* (SQ mean)	*L. infantum* (SQ mean)	*A. platys* (SQ mean)	*L. infantum* (SQ mean)	*A. platys* (SQ mean)	*L. infantum* (SQ mean)	*A. platys* (SQ mean)
820634	–^b^	2.31 × 10^-1^	–	3.50 × 10^-2^	2.36 × 10^-3^	–	–	2.78 × 10^-2^	–	–	–	–
821717	–	–	2.68 × 10^-3^	–	1.50 × 10^-3^	–	–	–	–	–	–	6.2 × 10^-3^
828965	–	–	2.61 × 10^-3^	3.36 × 10^2^	5.70 × 10^-3^	3.52 × 10	–	2.75 × 10^-2^	–	–	–	–
821240	–	3.85 × 10^-2^	2.08 × 10^-3^	2.31 × 10^-1^	8.28 × 10^-3^	3.75 × 10^-2^	–	–	–	2.27 × 10^-2^	1.21 × 10^-3^	–
823993	–	–	3.43 × 10^-3^	–	3.57 × 10^-3^	–	–	–	–	–	–	–
42686	1.40 × 10^-3^	–	6.14 × 10^-4^	–	5.54 × 10^-3^	–	–	–	–	–	–	–
43077	–	1.68 × 10^-1^	–	2.91 × 10^-1^	3.79 × 10^-3^	–	–	1.88 × 10^-1^	–	2.73 × 10^-1^	–	–
42783	–	–	1.52 × 10^-3^	–	9.02 × 10^-3^	2.83 × 10^-2^	–	–	–	–	–	–
43055	–	–	2.70 × 10^-4^	–	5.88 × 10^-4^	–	–	–	–	–	–	–
42825^a^	–	–	–	–	–	–	–	–	–	–	–	–

^a^Negative control; ^b^qPCR negative for pathogen DNA

**Table 4:** Real-time PCR detection and quantification of *Leishmania infantum* and *Anaplasma platys* DNA in tissue samples from non-pregnant bitches. Starting Quantity (SQ) mean value of parasite load per PCR reaction

Dogs	Blood		Bone marrow		Skin		Uterus		Ovary	
	*L. infantum* (SQ mean)	*A. platys* (SQ mean)	*L. infantum* (SQ mean)	*A. platys* (SQ mean)	*L. infantum* (SQ mean)	*A. platys* (SQ mean)	*L. infantum* (SQ mean)	*A. platys* (SQ mean)	*L. infantum* (SQ mean)	*A. platys* (SQ mean)
809546	2.44 × 10^-3^	–	2.19 × 10^-2^	–	1.63 × 10	–	2.37 × 10^-1^	–	3.03 × 10^-2^	–
43117	2.41 × 10^-1^	–	4.93	–	8.99 × 10^-3^	–	7.51	–	3.09 × 10	–
42853	–^b^	–	–	2.25 × 10^-1^	–	3.29 × 10^-2^	–	3.91 × 10^-2^	–	–
43017	–	–	–	–	–	–	–	2.24 × 10^-1^	–	5.83 × 10^-2^
43115	–	–	2.01 × 10^-1^	1.35	2.24	–	2.32 × 10^-3^	2.05	–	1.55
31585	–	–	–	–	–	–	–	1.20 × 10^-1^	–	4.80 × 10^-2^
42641	–	–	–	7.80 × 10^-2^	–	–	2.17 × 10^-3^	6.80 × 10^-2^	–	5.95 × 10^−2^
31339	–	–	2.50 × 10^-3^	1.90 × 10^-1^	–	–	–	2.81 × 10^-1^	–	5.78 × 10^-2^
830178	–	–	1.20 × 10^-2^	–	2.59 × 10^-2^	–	–	1.90 × 10^-1^	–	1.88
31956^a^	–	–	–	–	–	–	–	–	–	–

^a^Negative control; ^b^qPCR negative for pathogen DNA

Sixteen out of 54 (29.6 %) tissues from pregnant bitches were qPCR positive for *A. platys*, including three uterine, two ovarian and one placental sample with parasite loads ranging from 6.2 × 10^-3^ to 2.73 × 10^-1^/PCR reaction (Tables 1 and 3). Of the foetal tissues samples, five tested positive for *A. platys* (*n* = 2 liver; *n* = 3 spleen) with parasite loads ranging from 2.83 × 10^-2^ to 1.65 × 10^-1^/PCR reaction (Table 2). Of the 45 tissue samples collected from non-pregnant bitches (Tables 1 and 4), seven uteri and six ovaries scored qPCR positive for *A. platys*, with parasite loads ranging from 6.8 × 10^-2^ to 2.05/PCR reaction (Tables 1 and 4).

Ten bitches (*n* = 6 pregnant; *n* = 4 non-pregnant) were shown to be co-infected with both pathogens (Tables 1, 3 and 4). Two uterine samples from non-pregnant bitches were qPCR positive for *L. infantum* and *A. platys*, with parasite loads ranging from 2.17 × 10^-3^ to 2.32 × 10^-3^/PCR reaction and from 6.8 × 10^-2^ to 2.05/PCR reaction, respectively (Table 4). Tissues from dogs selected as negative controls on the basis of cytological examination of whole blood and bone marrow were qPCR negative for both pathogens (Tables 3 and 4).

## Discussion

This study provides new data regarding vertical transmission of *L. infantum* and *A. platys* in naturally infected dogs. Based on the samples examined, vertical transmission of *L. infantum* from an infected bitch to the offspring could be less frequent during the first half of gestation (i.e. 25–35 days). Indeed, a previous study investigating vertical transmission of *L. infantum* in eight bitches and their 53 foetuses during late pregnancy (i.e. 50–60 days) showed that 32 % of foetuses and 49 % of the corresponding placentas were PCR positive for this pathogen [24]. In contrast, the detection of *L. infantum* DNA in only one placenta, moreover with a low parasite load (i.e. 1.21 × 10^-3^), supports our hypothesis that this pathogen is unlikely to be transmitted during this phase of gestation. However, based upon the qPCR findings from non-pregnant bitches, *L. infantum* may be able to spread to the ovary, uterus and blood, with the former tissues displaying a higher parasite load (i.e. 3.09 × 10) compared to the latter (i.e. 2.41 × 10^-1^). Nevertheless, albeit promising, our preliminary data needs confirming in studies including a significantly larger number of infected bitches.

The contrasting findings observed for *L. infantum* between pregnant and non-pregnant bitches might be related to the hormonal changes associated with pregnancy and the different stages of the reproductive cycle. Increased oestrogen concentration in non-pregnant bitches may cause hyperaemia of the uterus and ovary tissues [25], which in turn may have been responsible for the increase in pathogen concentration in these sites. Alternatively, the fact that the uterus and ovary tissues of pregnant bitches were negative for *L. infantum* could be associated to the high blood flow resistance that characterises the canine placenta throughout the first half of gestation, and that progressively decreases in conjunction with the development of the foetal and placental circulation throughout the second half of gestation [26].

In previous studies, DNA from *L. infantum* was detected from foetal tissue samples (i.e. bone marrow, liver and spleen) over the last ten days of pregnancy (i.e. 50–60 days) and in newborn puppies [7, 10, 12, 24, 27, 28], but not from the uteri of infected pregnant bitches [27]. The detection of *L. infantum* amastigotes mostly in lymphoreticular organs (e.g. spleen, bone marrow, lymph nodes and liver) of late-gestation foetuses or newborn puppies [24], could mirror the distribution of parasites in tissues and organs of adult dogs. Therefore, it is tempting to speculate that the lack of *L. infantum*-PCR positive samples from foetuses examined in the present study may be linked to the immature state of the foetal immune system in early gestation. Lymphocytic infiltration of the foetal lymph nodes and spleen becomes evident from 45 to 52 days of gestation, which temporally corresponds to the development of the bone marrow that, at this time, contains a large number of hematopoietic stem cells [29].

This study provides first evidence supporting the hypothesis that *A. platys* may be vertically transmitted from the pregnant bitch to the offspring. Indeed, liver, spleen and uterine, ovarian, and placental tissues of foetuses and mothers positive for this pathogen were also positive for *A. platys*, with comparable pathogen loads (i.e. 2.83 × 10^-2^ in foetuses and 2.78 × 10^-2^ in mothers). The presence of the marginal haemophagous zones in the dog placenta, filled with extravasated maternal blood [30] could justify the detection of *A. platys*-infected platelets.

The above factors may also justify the finding of a higher *A. platys* load (i.e. 2.05) than that of *L. infantum* (i.e. 2.32 × 10^-3^) in the uterus of co-infected non-pregnant bitches in this study. The possibility of vertical transmission of *A. platys* in dogs is in agreement with knowledge of this event occurring for *Anaplasma marginale* and *Anaplasma phagocytophilum* in cattle [31, 32], whereas the minimal data relative to *in utero* transmission of *A. phagocytophilum* in dogs [33] did not support transplacental transmission. Controlled laboratory studies involving puppies from bitches infected with *A. platys* should be addressed to better understand this transmission pattern. To mimic a natural infection, bitches should be infected with *A. platys* by blood transfusion or by a competent vector.

However, confirmatory studies on naturally infected pregnant bitches (both symptomatic and asymptomatic) in early (25–35 days) and late pregnancy (50–60 days) and on their foetuses, need to be carried out in order to confirm or confute this hypothesis.

## Conclusion

Based on the results of the present study, transmission of *L. infantum* from infected mothers to their offspring appears unlikely or less frequent during the first half of gestation, whereas vertical transmission may occur during the later stages of gestation. In contrast, vertical transmission of *A. platys* may occur during the early stages of gestation, and throughout its entire course. If confirmed, these findings will pose additional challenges for the development of disease control strategies in both endemic and non-endemic areas.
